# Pathway Analysis of Genetic Factors Associated with Spontaneous Preterm Birth and Pre-Labor Preterm Rupture of Membranes

**DOI:** 10.1371/journal.pone.0108578

**Published:** 2014-09-29

**Authors:** Antonio Capece, Olga Vasieva, Shireen Meher, Zarko Alfirevic, Ana Alfirevic

**Affiliations:** 1 Department of Women's and Children's Health, Institute of Translational Medicine, University of Liverpool, Liverpool, United Kingdom; 2 Institute of Integrative Biology, University of Liverpool, Liverpool, United Kingdom; 3 Department of Molecular and Clinical Pharmacology, Institute of Translational Medicine, University of Liverpool, Liverpool, United Kingdom; John Hunter Hospital, Australia

## Abstract

**Background:**

Pre-term birth (PTB) remains the leading cause of infant mortality and morbidity. Its etiology is multifactorial, with a strong genetic component. Genetic predisposition for the two subtypes, spontaneous PTB with intact membranes (sPTB) and preterm prelabor rapture of membranes (PPROM), and differences between them, have not yet been systematically summarised.

**Methods and findings:**

Our literature search identified 15 association studies conducted in 3,600 women on 2175 SNPs in 274 genes. We used Ingenuity software to impute gene pathways and networks related to sPTB and PPROM. Detailed insight in the defined functional ontologies clearly separated integrated datasets for sPTB and PPROM. Our analysis of upstream regulators of genes suggests that glucocorticoid receptor (NR3C1), peroxisome proliferator activated receptor γ (PPARG) and interferon regulating factor 3 (IRF3) may be sPTB specific. PPROM-specific genes may be regulated by estrogen receptor2 (ESR2) and signal transducer and activator of transcription (STAT1). The inflammatory transcription factor NFκB is linked to both sPTB and PPROM, however, their inflammatory response is distinctly different.

**Conclusions:**

Based on our analyses, we propose an autoimmune/hormonal regulation axis for sPTB, whilst pathways implicated in the etiology of PPROM include hematologic/coagulation function disorder, collagen metabolism, matrix degradation and local inflammation. Our hypothesis generating study has identified new candidate genes in the pathogenesis of PPROM and sPTB, which should be validated in large cohorts.

## Background

Preterm birth (PTB) is the leading cause of neonatal mortality and morbidity, with an incidence estimated between 5–12%, depending on the geographic region [1]–[3]. PTB may be the consequence of spontaneous preterm labor with intact membranes (sPTB), preterm prelabor ruptured membranes (PPROM), or indicated PTB (for fetal and maternal reasons). Although PPROM often leads to sPTB, it is recognized that PROM and sPTB can present as separate entities. A better understanding of the similarities and differences between the respective pathways would open new avenues for research and treatment.

Genetic predisposition to PTB has been demonstrated in numerous studies [4]–[6]. Among twin pregnancies, the genetic contribution to PTB is estimated to be approximately 30% [7], [8]. In singletons, genetic susceptibility to PTB is based on the evidence of familial aggregation [9], [10], measures of heritability [11]–[13], identification of disease-susceptibility genes and racial disparity in PTB rate [14]–[16] that may be related to differences in risk-predisposing allele frequencies [17]–[20]. PTB rates are higher in sisters of women with a history of PTB compared to their sisters-in-law (16% vs. 9%). Several studies have confirmed a two-fold increase in risk of PTB for black American women compared to white American, women even after controlling for socio-economic factors associated with PTB [21].

Single nucleotide polymorphisms (SNPs) are the most thoroughly investigated genetic markers in relation to PTB. Many SNPs in maternal or fetal genomes have been reported to be associated with spontaneous preterm birth, but often the results have not been replicated in subsequent studies [22]–[27]. New systems biology approaches allow integration of functional genomics related to a phenotype or a disease in a connectivity network [28], [29]. Such convergent genomics allow identification of factors that affect downstream or upstream regulation of many candidate genes. Multiple relevant factors may converge on a similar pathway or gene, or conversely, one upstream stress condition/transcription factor may be responsible for regulation of several different pathways. Pathways or genes identified through this process may not be detected in individual genetic association studies. This ‘convergent genomics’ approach has been undertaken in the area of preterm birth in a recent study evaluating the impact of genetics on gestation age at delivery. Pooling SNP data from the published genome wide association study (GWAS) on preterm birth [https://www.genevastudy.org/Publications] and data from the custom database on preterm birth (dbPTB) (http://ptbdb.cs.brown.edu/dbPTBv1.php) [30] the authors analysed genetic contribution to preterm birth in three gestation categories: less than 30 weeks, less than 34 weeks and less than 37 weeks, but they did not take into account phenotypic differences. To our knowledge, the comparative analysis of PPROM and sPTB, while taking ethnicity into account, has not yet been undertaken at this methodological level.

We have, therefore, performed a systematic literature review of studies that investigated genetic factors involved in PTB to assess whether maternal candidate genes and SNPs previously identified in association with preterm birth can be used to:

1) identify maternal genetic markers for stratification of preterm birth due to PPROM or to spontaneous PTB with intact membranes

2) to differentiate between and clarify pathophysiological mechanisms involved in spontaneous PTB with intact membranes and PPROM

## Methods

### Search strategy

We searched Medline (Ovid) from 1^st^ January, 1990 to 1^st^ January, 2013, ISI Web of Knowledge, SCOPUS and databases including the Preterm Birth Genetics Knowledge Base, PTB Gene (http://ric.einstein.yu.edu/ptbgene/index.html) [31], KEGG (www.genome.jp/kegg), DAVID (david.abcc.ncifcrf.gov), GO (www.geneontology.org/GO.database.shtml) and Ingenuity Pathway analysis (IPA) (http://www.ingenuity.com/products/ipa) for studies assessing maternal genetic polymorphisms in association with preterm birth. The publically accessible Database for Preterm Birth (dbPTB) (ptbdb.cs.brown.edu/dbPTBv1.php) is a web-based tool which contains genes, genetic variants and pathways involved in preterm birth and any additional information on related literature. The search terms used to identify studies included “preterm birth” OR “preterm labor” OR “PPROM” combined with “polymorphisms” OR “genetic” OR “genes”, restricted to human. We also searched reference lists of study reports and review articles. Studies published only as abstracts were also included. No language restrictions were applied.

### Selection of studies

Three review authors (AC, AA and SM) independently assessed studies for inclusion in the review, using the criteria above. Any discrepancies were resolved by discussion or by consulting an additional author (ZA).

### Inclusion criteria

Studies were included if they were assessing single nucleotide polymorphisms (SNPs) in women who had sPTB or PPROM before 37 weeks gestation. Definitions for sPTB and PPROM were used as described in the studies. Spontaneous preterm birth was defined as spontaneous preterm labor with intact membranes, followed by preterm birth before 37 weeks. Preterm PROM was defined as membrane rupture diagnosed by vaginal pooling of fluid and a positive nitrazine test, before 37 weeks gestation and at least one hour prior to the initiation of regular contractions. Only SNPs that were reported to be significantly associated (p value <0.05) with sPTB or PPROM were included in pathway analyses.

### Exclusion criteria

Studies were excluded if they investigated medically indicated preterm births for pregnancy complications such as pre-eclampsia, placenta praevia, fetal anomalies, and gestational diabetes, or if we were unable to separate the data for sPTB and PPROM. Review articles were also excluded.

### Data Extraction and management

Data were extracted from each included study, entered into Excel spreadsheets, and double checked for accuracy. To prevent bias from multiple publications from the same cohort, only the largest number reported and/or the latest manuscript was used to calculate population size. Data were collected on the gene name and official symbol, and the unique SNP identifier rs number, was obtained from the databases dbSNP, OMIM and PTB Gene. The ethnicity of the population was noted. The genotype and allele association was recorded and expressed in terms of p-value, for each SNP.

### Pathway analysis

We used the Ingenuity Pathway Analyses (IPA) (Ingenuity Systems, Inc., Redwood City, CA, USA) to examine biological networks and disease functions associated with SNPs in maternal genomes that were significantly associated with either sPTB or PPROM. The Ingenuity Pathway Analysis (IPA) software package (http://www.ingenuity.com) uses the Ingenuity Knowledge Base, a manually curated database which contains information from the published literature as well as many other sources, including several gene expression and gene annotation databases such as IntACT, BIND, MiPs et al. [32]. IPA also includes interaction data to assign genes to different groups and categories of functionally related genes. It measures the associations of genes that are entered into the software, termed “focus genes”, with other molecules, such as proteins, genes, cells, tissues, diseases or medications. Focus genes are defined in the Ingenuity Pathway Analysis as the important (associated or above a cut-off) genes which interact with molecules in the Global Molecular Network. A gene cannot be considered a focus gene if there are no known molecular interactions involving that gene in the Ingenuity Knowledge Database. Ingenuity calculates single p-values for the enrichment of each gene category using the Fisher's exact test, taking into consideration both, the total number of molecules from the analyzed data set and the total number of molecules linked to the same gene category in the Ingenuity Knowledge Base reference set. For each gene category, correction for multiple comparisons is calculated using the Benjamini-Hochberg method and corrected p values of enrichment are provided [32]. Graphical presentation of networks is available as well as a score for each network. The score represents an approximate interaction between the focus molecule and each network [33].

We have also used IPA to identify functionally related genes that correspond to specific canonical pathways from a collection of 200 well-characterised metabolic and cell-signaling cascade pathways manually curated by IPA scientists from journal articles, text books, and KEGG ligand database. The Fisher’s exact test is used to calculate the probability that the association between the genes in the dataset and the canonical pathway can be explained by chance alone. Finally, we used the IPA upstream regulator analysis to identify transcriptional factors that may control genes and their pathways identified in relation to preterm birth, and to further examine how they may regulate their targets in order to provide testable hypotheses for gene regulatory networks (http://www.ingenuity.com/wp-content/themes/ingenuitytheme/pdf/ipa/feature_highlight_upstream_downstream.pdf).

### Datasets

Maternal genes or SNPs found to be significantly associated with sPTB and PPROM were uploaded separately into the IPA analysis tool. We examined the lists of all genes investigated in the included studies and compared them with the genes that were associated with each condition for similarities/differences. We related the genes to the postulated pathophysiological mechanisms of sPTB and PPROM. The software analysis mapped these focus genes to biological networks and disease functions. Comparative analyses were carried out on IPA for the two groups, sPTB and PPROM. The data generated were compared with regards to: i) the number and type of biological networks involved in each group, ii) genes/molecules common between PPROM and sPTB, and molecules unique to each group, iii) differences in the top disease functions within the networks, iv) differences in the top disease functions in the canonical pathways and v) any differences in the top transcriptional regulators involved in PPROM compared to sPTB. The number of links between molecules eligible for each network has been set to 140, which is the maximum number of connections in any network allowed by the IPA software. Links relevant only for mammalian species are used to reconstruct networks.

## Results

**Figure 1:**
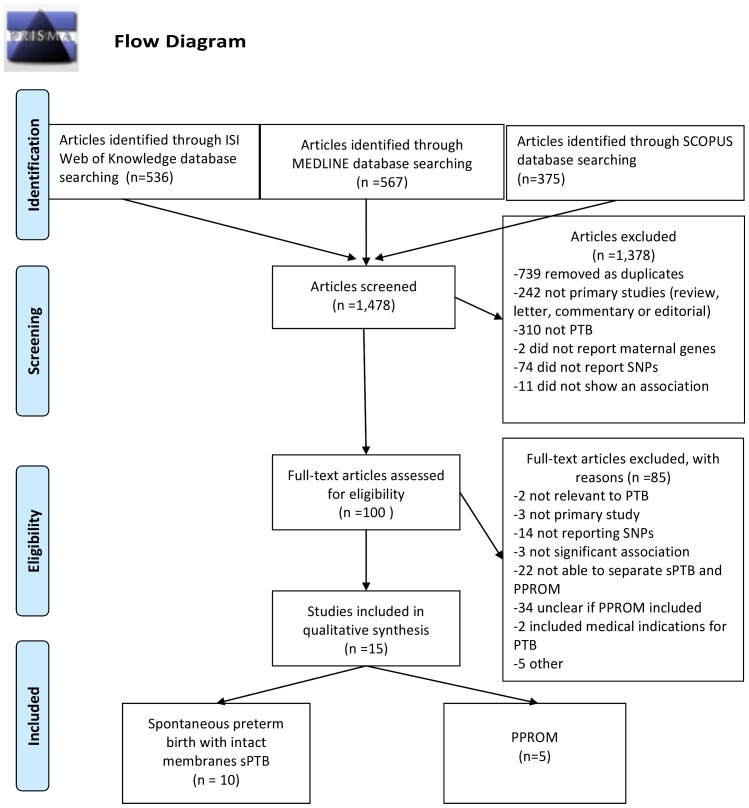
Literature search results.

A total of 1,424 reports were retrieved using our search strategy (**Figure 1**). Seven hundred and thirty nine duplicates were removed, two hundred and forty two studies were excluded after screening the titles and abstract because they were not primary studies, not related to preterm birth, did not report maternal genes or SNPs or were not associated with either sPTB or PPROM. Full text articles were retrieved for the remaining 100 potentially eligible reports (the list of all studies is available on request). Fifteen studies met the criteria and were included in the analyses (10 on sPTB with intact membranes [18], [34]–[42] and 5 on PPROM [27], [33], [43]–[47]. A total of 3,600 cases and controls matched for ancestry (White European, African American, South American (Latino) and Japanese) were included. Of these, a total of 878 women experienced PTB (N = 572 with sPTB and N = 306 with PPROM) and 2,722 women had term births defined as>37weeks.

A total of 2175 SNPs in 274 genes were investigated; 2169 SNPs in 266 genes were included in the studies on sPTB and 780 SNPs in 190 genes were genotyped in the studies on PPROM. 188 genes were investigated in relation to both sPTB and PPROM.

A total of 248 SNPs in 102 genes were found to be statistically significantly associated (p<0.05) with spontaneous preterm birth with intact membranes (sPTB), and 39 SNPs in 32 genes were found to be statistically significantly associated with PPROM (**Table 1** **)**. These genes were uploaded into the IPA for network analyses. Most of the genes analyzed for sPTB and PPROM phenotypes were studied in the Chilean population [33]. In addition, 30 genes from the significantly associated sPTB gene set were studied in African American population, but only in relation to sPTB and not PPROM [41] (Table S1).

**Table 1:** Focus genes used in Ingenuity Pathway Analysis.

**Focus genes PPROM**	**33 genes:** ACE, ANG, AGT, CD14, COL1A1, COL4A3, COL4A4, CSF1, CSPG2,F3, FGF4, IFNGR2, IGF1, IGF1R, IL1A, IL6R, LIPC, LPL, MMP1, MMP10,NOS2A, NOS3, PLAT, PTGER1, REN, TBXAS1, TIMP2, TNFA, TNFRSF6,TNFR2, TNR, TRL1, VWF.
**Focus genes sPTB**	**102 genes:** ACE, ADRB2, AGT, AGTR1, AP3M2, CCL2, CCL8, COL1A2,COL3A1, COL4A2, COL4A3, COL4A4, COL5A1, COL5A2, CRHBP, CRHR2,CTLA4, CYP19A1, CYP2D6, DHFR, EPHX1, EPHX2, F3, FGF1, F5, F7, GNB3,HSD17B7, HSPA1B, HSPA1L, HSPA4, HSPA6, HSPG2, HTR2A, IFNG, IGF1,IL10, IL10RA, IL10RB, IL12B, IL15, IL18, IL1A, IL1B, IL1R1, IL1R2, IL1RAP,IL1RN, IL2RA, IL2RB, IL4, IL4R, IL5, IL6, IL6R, IL8, KL, LTF, MMP1, MMP10,MMP16, MMP2, MMP3, MMP8, MMP9, MTHFD1, NAT1, NFKB1, NFKBIB,NOD2, NOS3, NR3C1, OXTR, PAFAH1B1, CBY1, PGRMC1, PLA2G4A, PLAT,POMC, PON1, PON2, PTGER3, PTGS1, REN, SCNN1A, SELE, SERPINE1,SLC6A4, TBXAS1, TEX12, THBS4, THPO, TIMP2, TIMP3, TLR2, TLR7, TNFR1,TNFR2, TSHR, UGT1A1, VEGFA, VEGFC.
**Focus genes common to PPROM & sPTB**	**16 genes:** ACE, AGT, COL4A3, COL4A4, F3, IGF1, IL1A, IL6R, MMP1, MMP10,NOS3, PLAT, REN, TBXAS1, TIMP2, TNFR2
**Focus genes unique to PPROM**	**17 genes** * **:** ANG, CD14, COL1A1, CSF1, CSPG2, FGF4, IFNGR2, IGF1R, LIPC,LPL, NOS2A, PTGER1, TNF, **TNFRSF6**, TNR, TLR1, VWF.
**Focus genes unique to sPTB**	**86 genes** ** **: ADRB2, AGTR1,** AP3M2, **CCL2,** CCL8, **COL1A2, COL3A1,** **COL4A2, COL5A1, COL5A2,** CRHBP, **CRHR2,** CTLA4, CYP19A1, CYP2D6,DHFR, EPHX1, EPHX2, FGF1, **F5, F7, GNB3,** HSD17B7, HSPA1B, HSPA1L,HSPA4, HSPA6, **HSPG2, HTR2A,** IFNG, **IL10, IL10RA,** IL10RB, **IL12B,** IL15,IL18, **IL1B, IL1R1, IL1R2,** IL1RAP, **IL1RN, IL2RA,** IL2RB, **IL4, IL4R, IL5, IL6,** **IL8,** KL, **LTF,** MMP16, **MMP2, MMP3, MMP8, MMP9,** MTHFD1, NAT1,NFKB1, NFKBIB, NOD2, NR3C1, **OXTR, PAFAH1B1,** CBY1, PGRMC1,PLA2G4A, POMC, **PON1,** PON2, **PTGER3, PTGS1,** SCNN1A, **SELE,** **SERPINE1,** SLC6A4, TEX12, **THBS4, THPO,** TIMP3, **TLR2,** TLR7, TNFR1,TSHR, UGT1A1, VEGFA, VEGFC.

Focus genes are defined in the Ingenuity Pathway Analysis as the important (associated or above a cutoff) genes which interact with molecules in the Global Molecular Network.

*The only gene from the list of 17 genes unique to PPROM that was not investigated in sPTB was TNFRSF6 (Highlighted in bold). All other genes were investigated in both, sPTB and PPROM, but were positive for PPROM only.

** From this list of 86 associated genes, 44 were investigated in sPTB only, but not in PPROM. Genes (N = 42) not validated for PPROM, are highlighted in bold.

### Ingenuity Pathway analysis

#### Network analysis

We performed the IPA analysis of the PPROM and integrated sPTB datasets based on ontology classification. Results from the comparative analyses between sPTB and PPROM are presented in Table S2. The focus genes uploaded for PPROM were mapped to 3 networks with scores ranging from 2.3 to 51, however, only one network reached a score above 3 (Network A). For sPTB, focus genes were mapped to 10 networks with scores ranging from 2 to 115, and only two networks had a score above 3 (Networks B and C). Each network included a number of “new” molecules (not from the input gene list) that were assigned to the network by the IPA algorithm (Network A = 111, Network B = 69, Network C = 123).

#### Function analyses

IPA algorithm identified the top ranked functions of these 3 networks: cellular movement and immune cell trafficking represented the first two functions in Network A and Network B, while the top ranked network function involved in Network C was cellular growth and proliferation. The top four molecular and cellular functions identified by IPA were exactly the same for the sPTB and PPROM. These were: i) cell to cell signaling and interaction, ii) cellular movement, iii) lipid metabolism and iv) small molecule biochemistry.

Although by general ontological description the data sets looked similar, more detailed insight in the defined functional ontologies has uncovered the distinct characteristics specific for each phenotype. sPTB appears to be associated with glucocorticoid signaling pathway with the following downstream affected genes: ADRB2, AGT, CCL2, HSPA4, HSPA6, HSPA1B, HSPA1L, IFNG, IL5, IL6, IL8, IL10, IL1B, IL1R2, IL1RN, IL4, MMP1, NFKB1, NFKBIB, NR3C1, POMC, SELE and SERPINE1. Apart from glucocorticoid signaling, the identified markers were clearly inflammatory, matrix degrading or collagen metabolism related markers along with NF-kB related molecules, which indicate an inflammatory component. The only two genes from the list representing glucocorticoid signaling pathway that overlap with PPROM are AGT and MMP1. AGT encodes angiotensinogen, a precursor of angiotensin involved in controlling arterial pressure, which has been associated with preterm-birth regardless of etiology [46]. MMP1 encodes matrix metalloproteinase 1 which is higher in women delivering preterm compared to those delivering at term [48].

In contrast, two genes, TNF and NOS2A, were only detected in PPROM but not sPTB. TNF encodes tumor necrosis factor α, a pro-inflammatory cytokine found in the amniotic fluid and in blood of mothers and fetuses with PTB [49]. NOS2A encodes nitric oxide synthase 2A, a P450 type protein which is found in the foeto-placental unit that may be leading to the reduced placental blood flow and increased resistance in the foeto-maternal circulation [50]. In addition, NOS2s are important in cytokine signaling and innate immunity and are involved in an antimicrobial pathway.

Regarding lipid metabolism, sPTB and PPROM are characterized by different functions. sPTB shows specific functional enrichment in eicosanoid metabolic pathways and functions. Eicosanoids are signaling molecules derived from fatty acids, involved in inflammation and immunity (Table S3). In contrast, in PPROM dataset ‘Lipid metabolism’ ontological grouping, metabolism of eicosanoids does not present at a high significance level. Although the PPROM list of functional annotations is shorter and shows less significant p-values compared with sPTB, it has a distinct enrichment of functions related to lipid metabolism (triglycerides and fatty acids) and the lipid metabolic disorders such as hyperlipidemia, hypercholesterolemia (genes represented include HDL, LIPC and FAS) (Table S3).

There are also differences in connective tissue functions between sPTB and PPROM, such as altered fibroblast proliferation (TNFA and IGF1vs NOS2 and NOS3), blood pressure and kidney dysfunction (PLAT). In addition, connective tissue disorders have different components in sPTB and pPROM. In PPROM, structural and mechanical tissue damage-related functions (IGF1, IL1A, TNF, TNFRSF1B) and weak autoimmune component (ACE, ANG, CSF1 (includes EG:12977), IL1A, IL6R, MMP1 (includes EG:300339), MMP10, NOS2, TNF, TNFRSF1B) are identified (Table S4). In sPTB in contrast, the cell-mediated-immune response was one of the dominating categories. A strong autoimmune component is represented with the large number of immune-related genes (ACE, ADRB2, CCL2, CCL8, COL3A1, CTLA4, CYP19A1, DHFR, EPHX2, HSPA1A/HSPA1B, HSPA1L, IFNG (includes EG:15978), IL10, IL10RA, IL12B, IL18 (includes EG:16173), IL1A, IL1B, IL1R1, IL1R2, IL1RN, IL2RA, IL2RB, IL4 (includes EG:16189), IL4R, IL5, IL6, IL6R, IL8, LTF, MMP1 (includes EG:300339), MMP10, MMP16, MMP2, MMP3, MMP8, MMP9, NFKB1, NOD2, NR3C1, POMC, PTGS1, SELE (includes EG:20339), SLC6A4, TIMP3, TLR2, TLR7, TNFRSF1A, TNFRSF1B, VEGFA) (Table S4).

#### Disease analysis

The top ranked disease functions involved with PPROM were inflammatory processes while the top disease functions involved with sPTB were connective tissue disorders (Tables S2 and S4). Although both sPTB and PPROM have inflammatory and connective tissue disorders among the top 5 ranked diseases, their inflammatory categories vary greatly as demonstrated in Table S5. sPTB is associated with rheumatic disease and autoimmune diseases (including neurological and psychiatric diseases), endometriosis and particularly strongly linked with infection (bacterial, parasitic or viral origin). Cell mediated immune response in sPTB is associated with the following functions: IFNG (includes EG:15978), IL10, IL12B, IL15 (includes EG:16168), IL1B, IL2RA, IL2RB, IL4 (includes EG:16189), IL6, TLR7 for T cells homeostasis, and IFNG (includes EG:15978), IL12B, IL1B, IL2RA, IL2RB, IL4 (includes EG:16189), IL6, TLR7 for differentiation of helper cells.

In PPROM however, inflammation component was related mainly to local organ inflammation with the highest scores p = 10^−14^ to p = 10^−7^ with the functions of ACE, AGT, CD14, COL4A3, COL4A4, FAS, IFNGR2, IGF1, MMP1 (includes EG:300339), NOS2, NOS3, REN, TNF. Further diseases linked to PPROM were nephritis or glomerulonephritis (ACE, COL4A3, COL4A4, IGF1, NOS2, REN, TNF) (Table S5). In addition, PPROM inflammatory response is supported mainly by macrophages and especially phagocyte migration, activation and proliferation (AGT, CSF1 (includes EG:12977), FAS, TIMP2 (includes EG:21858), TNF, IL6R, CD14, TNFRSF1B). These have been previously associated with early onset sepsis in very low birthweight infants [51], and chorioamnionitis-associated preterm delivery [52]. Cell mediated immune response specific for PPROM also includes differentiation of helper T lymphocytes (P = 1.35×10^−12^), T cell homeostasis (p = 5.04×10^−11^), differentiation of T lymphocytes (p = 6.53×10^−11^).

Similarities between sPTB and PPROM include the ACE and AGT genes which might be responsible for hematologic/coagulation function disorders linked with markers (STAT1, MMP1, TNF, IGF1, MMP9). They are involved in collagen metabolism and matrix degradation, an effector of PPROM [53]–[57]. Functional SNPs in these genes and their action may be exaggerated by coagulopathies (ACE, AGT) or by apoptosis (STAT1) [58]. In PPROM, an increased synergistic effect on vasculature and heart dynamics may be associated with several additional genes TNFA, IGF1, NOS2 and NOS3.

Body mass index (BMI) was associated with both sPTB and PPROM and also two top canonical pathways “Hepatic fibrosis/hepatic stellate cell activation” and “Atherosclerosis Signaling”, although the content of the functions grouped by these categories was different as mentioned above. Both, low and high BMI has been associated with PTB previously [59].

#### Network analysis

After analysing individual functions and diseases related to sPTB and PPROM, we overlayed the networks reconstructed from the functions with the corresponding ontological categories to illustrate the dominant associations. We reconstructed dense networks with connected functions, which were not present in the starting lists of genes. We also refined one network for each condition. All molecules in the three networks which showed a significant association (score>3) are presented in Table S6.

**Figure 2:**
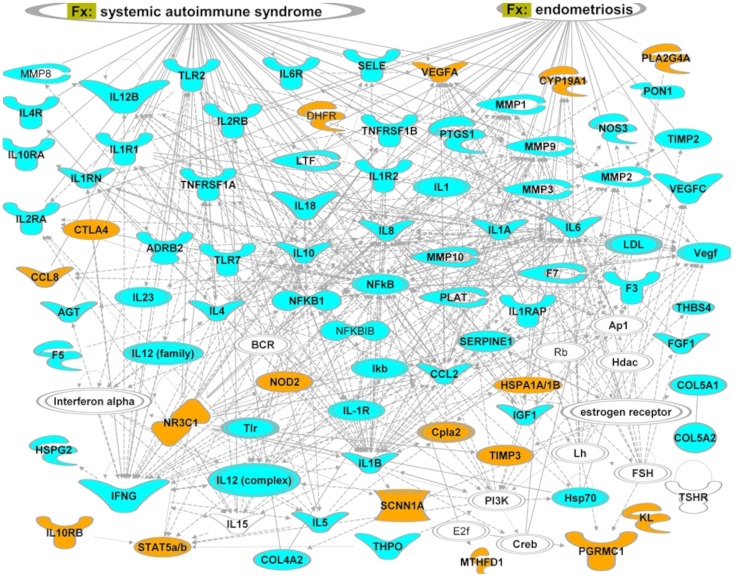
Top ranked gene network reconstructed from genes associated with sPTB (blue and orange blocks). Genes unique for the African American dataset, not studied in relation to pPROM condition are shown in orange. Links projected from ‘Functions and diseases’ (Fx) categories of IPA’s knowledge database are shown in oval shapes at the top of the figure. Lines correspond to functional (arrows) or physical interactions (lines) between proteins. An arrow indicates a regulatory vector. Solid lines imply direct relationships between proteins, dotted lines imply indirect interactions. Relationships include post-translational modifications, transcription regulation, proteolysis or co-expression. Different node shapes represent the following: complexes or unknown (NfkB), cytokines and growth factors (IL8), enzymes (DHFR), peptidases (MMP1), transcription regulators (NFKB1), nuclear receptors (NR3C1), channels (SCNN1A).

Figure 2. illustrates connectivity between genes associated with sPTB and their relation to few dominant functional categories. We used IPA design tool to re-arrange the nodes in each network so that the functions associated with each category are located close to the corresponding category labels. Only the NFκB TF is associated with all the main categories and with the majority of the genes. A number of interleukins and growth factors specific for sPTB: IL18, IL12B, IL8, IL4, IL10, PTGS1 or shared between two subtypes: F3, TNFRSF1B, TNFRSF1A, are linked to all functional categories.

**Figure 3:**
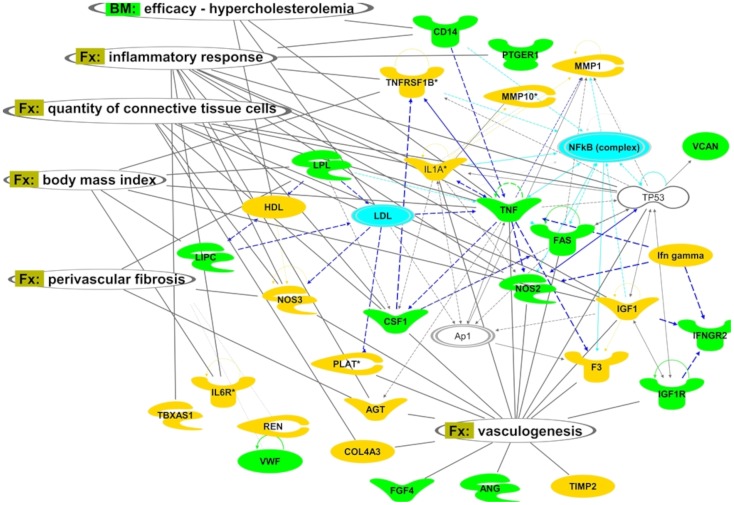
Top ranked gene network reconstructed from genes associated with PPROM (green and blue blocks). Green color represents pPROM specific functions, blue- genes associated with both sPTB and pPROM and yellow-functions specific for sPTB. Ovals- links projected from ‘Functions and diseases’ (Fx) and ‘Biomarkers’ (Bm) categories of IPA’s knowledge database. Lines correspond to functional (arrows) or physical interactions (lines) between proteins. An arrow indicates a regulatory vector. Solid lines imply direct relationships between proteins, dotted lines imply indirect interactions. Relationships include post-translational modifications, transcription regulation, proteolysis or co-expression. The blue lines indicate the NfkB area of influence. Different node shapes represent the following: complexes or unknown (an example is NfkB), cytokines and growth factors (as for CSF1), enzymes (NOS3), peptidases (MMP1), transcription regulators (TP53).

Top ranked PPROM-associated functional network is shown in Figure 3. Vasculogenesis can be observed in association with PPROM-specific functions. Blood pressure category, although more profound for the PPROM set, is also associated with the sPTB list of genes.

#### Upstream transcriptional regulation

**Figure 4:**
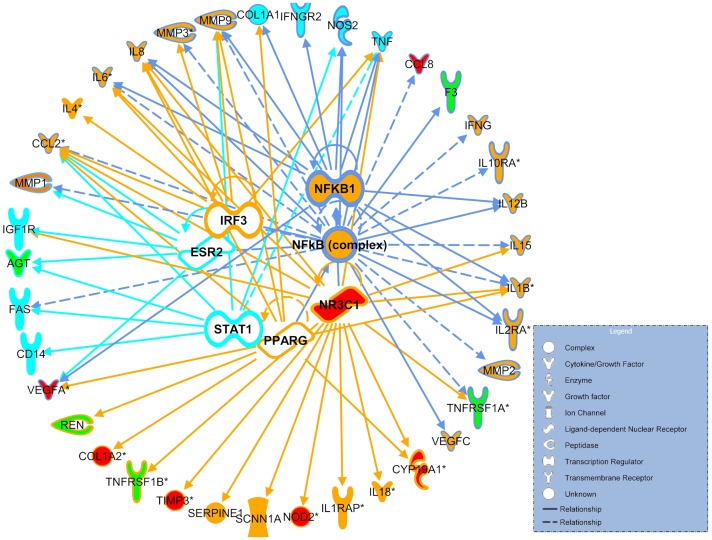
Top ranked upstream regulators (inner circle) suggested by IPA for preterm birth-associated genes (outer circle). Orange blocks-genes unique for sPTB, turquoise blocks- genes unique for pPROM and green blocks correspond to genes associated with both sPTB and pPROM. Functions of the main transcription regulators are presented with different colour lines. Blue lines represent both sPTB and pPROM regulators (NFkB1, NFkB complex), while turquoise and orange lines represent mainly pPROM (PPARG, IRF3 and NR3C1) and sPTB (STAT1 and ESR2), respectively. Genes with SNPs studied only in relation to sPTB in African American population are presented in red. Regulation by NFkB is shown in blue lines, IRF3 or NR3C1 in orange, ESR2 or STAT1 in turquoise. Arrows correspond to transcription regulation and lines to physical interactions between proteins. Solid lines imply direct and dotted lines imply indirect transcription regulation. Different node shapes and their functions are presented in the legend.

We used IPA upstream regulator analysis to predict the potential TFs responsible for differential gene expression in sPTB and pPROM. Regulatory interactions for the sPTB and pPROM gene lists suggested by the IPA largely overlap, although the association with sPTB shows lower p-values. The NfKB complex is highly ranked for both, SPTB and PPROM through the network connectivity, therefore the fraction of genes common for preterm birth conditions is likely regulated by the inflammatory TF NFκB. Our predicted TFs are shown in Figure 4 together with the downstream genes that they may regulate. Nearly all PPROM-specific genes are linked to a signal transducer and activator of transcription 1 (STAT1). STAT1 is a 91 kD protein, member of the STAT family of transcription factors originally identified as the mediators of the cellular response to interferon alpha (IFN) and estrogen receptor 2 (ESR2). Genes unique to sPTB are regulated mainly by three TFs; glucocorticoid receptor (NR3C1), peroxsome proliferator activated receptor γ (PPARG) and interferon regulating factor 3 (IRF3). Interestingly, the NR3C1 polymorphysm shown to be associated with sPTB, is included in the dataset and appears in the top network reconstructed for sPTB.

## Discussion

Based on our analyses, we propose autoimmune/hormonal regulation axis for sPTB, whilst pathways implicated in the etiology of PPROM include hematologic/coagulation function disorder, collagen metabolism, matrix degradation and local inflammation. We could clearly separate integrated datasets/pathways for sPTB and PPROM and identified genes encoding transcription factors specific for sPTB (NR3C1, PPARG and IRF3) and PPROM (STAT1 and ESR2), or common for both subtypes (NFκB complex).

Earlier genetic studies achieved only limited success in explaining heritability of PTB. They have been hampered by a relatively small number of participants with variable ethnic background, lack of well characterized phenotypes, lack of replication of genetic associations in subsequent studies and up until recently, lack of unbiased genome-wide association studies. It is likely that variants of many genes contribute to the final PTB with only a modest effect. These are difficult to identify and will often be missed in small scale association studies. In addition, these candidate gene association studies are not designed to investigate gene-gene interactions. By integrating the genomic data on a large number of individuals into networks, we have overcome the low statistical power of individual studies. The analysis also permitted inclusion of different ethnic groups. Our network analysis has allowed us to expand gene selection and include some, as yet unexplored candidates. It is noteworthy that potential for false positive associations is usually small in the core network connections where several interconnected functions may suggest potential targets which could be subsequently validated. In addition, the IPA software allows correction for false positive discovery when multiple comparisons are involved.

We included the data from multiple gene/polymorphisms association analyses related to PTB in different ethnic groups. For sPTB, we included three populations: 1) South American (Chilean), 2) African-American and 3) Caucausian. However, data for African-American and Caucasian women with PPROM were not available. Interestingly, we found a difference in functional description among different ethnicities. As shown in Figure 2, only data for the African-American ethnic group point to a role of hormonal regulation in preterm labor. It remains unclear whether the hormonal regulation of sPTB is indeed ethnicity specific, although large variability in the incidence of preterm labor in different ethnicities support the association [17], [20], [60]. We hypothesized that although different ethnic groups may bear different polymorphisms in relation to PTB, they are tightly linked to the network of ethnicity invariant functions associated with sPTB. Hormonal deregulation may comprise an upstream or a downstream component in the chain of events leading to sPTB. It will be interesting to find out if an ethnic group which is at higher risk for PTB, has higher frequency of specific polymorphisms in a number of genes (Cpla2, Vegf, VEGFA, CYP19A1, HSPA1A and A1B, PGRMC1) in the proximity of LH and FSH functions (Figure 2). We recognized that the hormone-regulatory sub-network which includes FSH, estrogen, progesterone, LH and their connected functions is central to sPTB. This was also confirmed in African Americans (Figure 2) where FSH is linked strongly with the network reconstructed from the sPTB associated genes. Our findings are in keeping with a recent phylogenetic analysis of potential association between genes involved in human birth acceleration and preterm birth risk [61]. The study revealed a pivotal role of the defined ‘hormonal’ sPTB network. Screening of 8,400 SNPs in 150 human accelerated genes for association with preterm birth in 165 Finnish preterm and 163 control mothers showed that 8 of the 10 most significant SNPs were identified in the FSHR gene. The hormone-regulatory component of the sPTB gene group network connects all other sPTB-associated functions in the network, indicating that this may be crucial for the upstream pathways such as the infection/inflammation pathway.

The most densely connected to sPTB is the inflammatory/infection related network that can be present upstream as well as downstream of associated hormonal perturbations. The predicted TFs, such as NR3C1 and PPARG are known modulators of hormonal regulation of immunity and lipid metabolism and have been implicated in preterm birth infants previously [62]–[65]. We identified PPARG as one of the leading regulators for sPTB related to modulation of expression of lipid metabolism genes and hormone regulation functions.

Endogenous glucocorticoids influence fetal development and regulate glucose and fat metabolism, cardiovascular system and immune functions. Several polymorphisms in the glucocorticoid receptor gene (NR3C1) are linked to altered function of glucocorticoid receptor. SNPs may increase glucocorticoid sensitivity *in vivo*, and have been associated with birth weight in preterm neonates [62], [63].

STAT1 which is identified as PPROM-specific regulator, belongs to a family of transcription factors. STAT1 was initially identified as an interferon α (IFNα) mediator and a major component of the cellular response to IFNγ [66]. STAT1 regulates the immune system, cell differentiation, cell growth inhibition and apoptosis [67]. It is interesting that STAT1 was recently shown to be associated with renin-angiotensin system [68] and may be a regulator of the genes associated with a ‘blood pressure’ category specifically enriched in the PPROM-associated dataset. STAT1 is also involved in the negative regulation of angiogenic factors such as matrix metaloproteinases (MMP9) [67]. MMPs play a central role in cell proliferation, migration, differentiation, angiogenesis, apoptosis and host defences. The interaction of STAT1 with other transcription factors varies with the cellular system and it can induce the constitutive expression of a subset of genes involved in immune regulation. Mutations in the STAT1 gene leading to its reduced activity are associated with infectious disease. In contrast, a high level of expression of STAT1 stimulates the TNF-α apoptotic pathways and inhibits NF-κB [69]. Increasing concentration of TNFα in umbilical cord was associated with an increased risk of preterm birth [70].

Another transcription regulator linked to PPROM is estrogen receptor 2 (ESR2), a member of the family of estrogen receptors and superfamily of nuclear receptor transcription factors. Enhanced estrogen receptor activity is involved in the proinflammatory cascade leading to parturition. Furthermore, estrogen receptor activation facilitates labor by enhancing transcription of genes encoding uterine contraction-associated proteins including oxytocin and COX-2, which catalyzes the production of prostaglandins [71].

Distinct functions associated with the studied gene group and network connectivity of predicted TFs are indicative of diverse etiology for PPROM and sPTB. A relatively small number of studies and genes was included in the analyses of PPROM, therefore, further work on PPROM is required using an unbiased genome wide approach to elucidate potential novel mechanisms.

One of the weaknesses of this study is reliance on candidate gene association studies. Lack of GWAS studies specific to sPTB and pPROM results in biases in candidate gene selection. As many of the authors of candidate gene studies have been involved in infection/inflammation area of research, that pathway dominates in gene selection. However, the unbiased GWAS approaches such as the GENEVA GWAS study, failed to generate any genome-wide significant results [30], [72] potentially because spontaneous PTB cases were analysed together with all indicated PTB cases. Future GWAS studies should eliminate the biases in candidate gene selection and examine further diverse etiology of sPTB and pPROM. In addition, gene-gene and gene-environment interactions cannot be assessed through IPA analysis. These, together with comorbidity such as coagulopathies and collagen disorders, and their interaction with inflammatory triggers that may produce discrete phenotypes, will have to be incorporated into clinical trial design and data collection of future studies.

We did not control for methodological quality of included studies. It was evident that quality of data from different studies varied; while comprehensive information on a large number of markers was available for Chilean [33] and African American groups [39], in Caucasians, only limited information was available from several small association studies often reporting a single gene analysis. Several authors did not report on quality control processes, and did not include information on genotype call frequency, validation cohorts or statistical power calculations. In addition, no formal genetic analysis has been conducted in these studies to test for population stratification. Future work should include unbiased whole genome approaches in large, phenotypically well characterized cohorts of women with PTB and controls matched for ancestry [73]. In order to calculate the required number of cases and controls in a genome-wide association study, which would allow to detect clinically relevant difference in allelic frequencies (OR≥3), approximately 350 PTB cases and 1400 controls would be required to achieve 80% power. We assumed an additive model of inheritance, 1∶4 ratio of cases: controls and genome wide significance (p = 1.00E-08) for alleles at>5% frequency. Such numbers of participants can be achieved only through international collaborations on PTB.

In conclusion, we predicted transcriptional regulators that may be related to etiology of PPROM and sPTB, and identified genetic markers that may help in the assessment of their potential risk factors. Heterogeneity in the natural history of preterm birth exists and therefore, a one-size-fits-all prevention strategy may not be appropriate. Prevention of preterm birth requires more research into the etiology of preterm birth subtypes and this study has contributed to our understanding of the molecular mechanisms and regulation of genes/pathways implicated in PPROM and sPTB. Our hypothesis generating study has identified several new candidate genes and their potential role in the pathogenesis of PPROM and sPTB, which should be explored further and validated in carefully planned experiments with large numbers of patients. The study represents the first step and foundation for future research, which will be conducted through access to the international biorepositories of blood or tissues from large numbers of pregnant women with well-defined and standardized phenotypes.

## Supporting Information

Table S1 **A list of genes (N = 30) associated with spontaneous preterm birth studied in the African American population [Velez et al.].**
(DOC)

Table S2 **Comparative IPA analyses of sPTB and PPROM.**
(DOC)

Table S3 **Comparison of pathways and functions in the ‘Lipid metabolism’ ontological grouping for PPROM and sPTB.** The pathways are ordered according to the p-value, from lowest p-value in each phenotype.
(DOC)

Table S4 **Connective Tissue disorders in PPROM and sPTB.** Diverse lists of disorders are shown for two phenotypes.
(DOC)

Table S5 **Inflammatory response in PPROM and sPTB.** Diverse lists of responses are shown for two phenotypes.
(DOC)

Table S6 **Comparison of molecules/genes in networks.** All molecules (focus genes (in bold) and new molecules identified by IPA) in each of the significant networks. Molecules common to the PPROM and sPTB networks (B and C) and those that are unique to PPROM (in neither of the two sPTB networks) are listed.
(DOC)
