# Supplementary material for: Pathway Analysis of Genetic Factors Associated with Spontaneous Preterm Birth and Pre-Labor Preterm Rupture of Membranes
Source: PLoS One. 2014 Sep 29;9(9):e108578. doi: 10.1371/journal.pone.0108578 (PMC4181300; doi:10.1371/journal.pone.0108578)
Supplement: Table S1 — A list of genes (N = 30) associated with spontaneous preterm birth studied in the African American population [Velez et al.]. (DOC) [file pone.0108578.s001.doc]

**Supplementary Table 1.** A list of genes (N=30) associated with spontaneous preterm birth studied in the African American population [Velez et al.]

| **Gene** | **Function** | **Network** |
| --- | --- | --- |
| **Symbol** | **Entrez Gene Name** |  |
| AP3M2 | adaptor-related protein complex 3, mu 2 subunit | 1 |
| CBY1 | chibby homolog 1 (Drosophila) | 2 |
| CCL8 | chemokine (C-C motif) ligand 8 | 1 |
| CRHBP | corticotropin releasing hormone binding protein |  |
| CTLA4 | cytotoxic T-lymphocyte-associated protein 4 | 1 |
| CYP19A1 | cytochrome P450, family 19, subfamily A, polypeptide 1 | 1 |
| CYP2D6 | cytochrome P450, family 2, subfamily D, polypeptide 6 |  |
| DHFR | dihydrofolate reductase | 1 |
| EPHX1 | epoxide hydrolase 1, microsomal (xenobiotic) | 2 |
| EPHX2 | epoxide hydrolase 2, cytoplasmic |  |
| HSD17B7 | hydroxysteroid (17-beta) dehydrogenase 7 |  |
| HSPA1A/HSPA1B | heat shock 70kDa protein 1A | 1 |
| HSPA1L | heat shock 70kDa protein 1-like |  |
| HSPA4 | heat shock 70kDa protein 4 | 2 |
| HSPA6 | heat shock 70kDa protein 6 (HSP70B') | 2 |
| IL10RB | interleukin 10 receptor, beta | 1 |
| IL15 (includes EG:16168) | interleukin 15 | 1 |
| KL | klotho | 1 |
| MTHFD1 | methylenetetrahydrofolate dehydrogenase (NADP+ dependent) 1, methenyltetrahydrofolate cyclohydrolase, formyltetrahydrofolate synthetase | 1 |
| NAT1 | N-acetyltransferase 1 (arylamine N-acetyltransferase) |  |
| NOD2 | nucleotide-binding oligomerization domain containing 2 | 1 |
| NR3C1 | nuclear receptor subfamily 3, group C, member 1 (glucocorticoid receptor) | 1 |
| PGRMC1 | progesterone receptor membrane component 1 | 1 |
| PLA2G4A | phospholipase A2, group IVA (cytosolic, calcium-dependent) | 1 |
| POMC | proopiomelanocortin | 2 |
| PON2 | paraoxonase 2 |  |
| SCNN1A | sodium channel, non-voltage-gated 1 alpha subunit | 1 |
| SLC6A4 | solute carrier family 6 (neurotransmitter transporter, serotonin), member 4 | 4 |
| TEX12 | testis expressed 12 |  |
| TIMP3 | TIMP metallopeptidase inhibitor 3 | 1 |
| TLR3 | toll-like receptor 3 |  |
| TSHR | thyroid stimulating hormone receptor | 1 |
| UGT1A1 | UDP glucuronosyltransferase 1 family, polypeptide A1 | 2 |
| VEGFA | vascular endothelial growth factor A | 1 |
