# Supplementary material for: Pathway Analysis of Genetic Factors Associated with Spontaneous Preterm Birth and Pre-Labor Preterm Rupture of Membranes
Source: PLoS One. 2014 Sep 29;9(9):e108578. doi: 10.1371/journal.pone.0108578 (PMC4181300; doi:10.1371/journal.pone.0108578)
Supplement: Table S2 — Comparative IPA analyses of sPTB and PPROM. (DOC) [file pone.0108578.s002.doc]

**Supplementary Table 2.** Comparative IPA analyses of sPTB and PPROM

|  | **PPROM** | **sPTB** |
| --- | --- | --- |
| Number of networks | 3 | 10 |
| network score (range) | 2 TO 51 | 2 TO 115 |
| Number of significant networks (criteria score > 3) | 1 | 2 |
|  | **Network A** | **Network B** |
| Score | 51 | 115 |
| Focus gene | 29 | 71 |
| New molecules | 111 | 69 |
| **TOP NETWORK FUNCTIONS** | 1) Cellular movement | 1) Cellular movement |
|  | 2) Immune cell trafficking | 2) Immune cell trafficking |
|  | 3) Cell to cell signalling and interaction | 3) Connective tissue disorders |
|  |  |  |
|  |  | **Network C** |
| Score |  | 13 |
| Focus gene |  | 17 |
| New molecules |  | 123 |
| **TOP NETWORK FUNCTIONS** |  | 1)Cellular growth and proliferation |
|  |  | 2) Cancer |
|  |  | 3) Cellular death |
|  |  |  |
| **TOP DISEASES** | 1) Inflammatory response | 1) Connective tissue disorders |
|  | 2) Neurological disease | 2) Inflammatory disease |
|  | 3) Psychological disease | 3) Skeletal and muscular disease |
|  | 4) Connective tissue disorders | 4) Immunological disease |
|  | 5) Organism injury and abnormalities | 5) Cardiovascular disease |
|  |  |  |
| **TOP CANONICAL PATHWAYS** | 1) Hepatic Fibrosis/ Hepatic Stellate Cell Activation | 1) Hepatic Fibrosis/ Hepatic Stellate Cell Activation |
|  | 2) Atherosclerosis Signaling | 2) Atherosclerosis Signaling |
|  | 3) LXR/RXR Activation | 3) Role of macrophages, Fibroblasts and Endothelial Cells in Rheumatoid Arthritis |
|  | 4) IL-6 Signaling | 4) Glucocorticoid Receptor Signaling |
|  | 5) Role of Osteoblast, Osteoclasts and Chondrocytes in Rheumatoid Arthritis | 5) T Helper Cell UNIQUEiation |
