# Supplementary material for: Pathway Analysis of Genetic Factors Associated with Spontaneous Preterm Birth and Pre-Labor Preterm Rupture of Membranes
Source: PLoS One. 2014 Sep 29;9(9):e108578. doi: 10.1371/journal.pone.0108578 (PMC4181300; doi:10.1371/journal.pone.0108578)
Supplement: Table S3 — Comparison of pathways and functions in the ‘Lipid metabolism’ ontological grouping for PPROM and sPTB. The pathways are ordered according to the p-value, from lowest p-value in each phenotype. (DOC) [file pone.0108578.s003.doc]

**Supplementary Table 3.** Comparison of pathways and functions in the ‘Lipid metabolism’ ontological grouping for PPROM and sPTB. The pathways are ordered according to the p-value, from lowest p-value in each phenotype.

| **PPROM** |  | **sPTB** |  |
| --- | --- | --- | --- |
| **Functions Annotation** | **p-Value** | **Functions Annotation** | **p-Value** |
| synthesis of eicosanoid | 6.22E-21 | synthesis of fatty acid | 7.48E-10 |
| synthesis of prostaglandin | 1.84E-20 | quantity of steroid | 2.37E-09 |
| synthesis of prostaglandin E2 | 2.29E-17 | synthesis of lipid | 5.12E-09 |
| synthesis of lipid | 1.05E-16 | fatty acid metabolism | 9.64E-09 |
| concentration of eicosanoid | 6.00E-15 | synthesis of prostaglandin | 3.08E-08 |
| concentration of fatty acid | 1.07E-14 | concentration of lipid | 1.33E-07 |
| concentration of prostaglandin | 3.09E-11 | concentration of triacylglycerol | 1.43E-07 |
| concentration of lipid | 4.48E-11 | synthesis of prostaglandin E2 | 3.49E-07 |
| concentration of prostaglandin E2 | 1.11E-09 | metabolism of acylglycerol | 2.12E-06 |
| synthesis of prostaglandin D2 | 1.11E-09 | hydrolysis of triacylglycerol | 6.37E-06 |
| release of lipid | 2.86E-09 | synthesis of prostaglandin D2 | 6.37E-06 |
| quantity of steroid hormone | 1.00E-07 | hydrolysis of phospholipid | 8.92E-06 |
| synthesis of 6-keto-prostaglandin F1 alpha | 1.00E-07 | hydrolysis of triolein | 1.59E-05 |
| quantity of steroid | 1.38E-07 | synthesis of androstenedione | 1.59E-05 |
| mass of fat | 4.96E-07 | release of lipid | 2.27E-05 |
| secretion of lipid | 1.24E-06 | concentration of cholesterol | 2.91E-05 |
| concentration of corticosterone | 1.60E-06 | hydrolysis of 1,2-dipalmitoylphosphatidylcholine | 3.17E-05 |
| synthesis of thromboxane B2 | 1.60E-06 | metabolism of triacylglycerol | 4.52E-05 |
| release of eicosanoid | 2.86E-06 | catabolism of triacylglycerol | 7.90E-05 |
