# Supplementary material for: Pathway Analysis of Genetic Factors Associated with Spontaneous Preterm Birth and Pre-Labor Preterm Rupture of Membranes
Source: PLoS One. 2014 Sep 29;9(9):e108578. doi: 10.1371/journal.pone.0108578 (PMC4181300; doi:10.1371/journal.pone.0108578)
Supplement: Table S4 — Connective Tissue disorders in PPROM and sPTB. Diverse lists of disorders are shown for two phenotypes. (DOC) [file pone.0108578.s004.doc]

**Supplementary Table 4. Connective Tissue disorders in PPROM and sPTB**

Diverse lists of disorders are shown for two phenotypes.

| **PPROM** |  | **sPTB** |  |
| --- | --- | --- | --- |
| **Functions Annotation** | **p-Value** | **Functions Annotation** | **p-Value** |
| damage of connective tissue | 1.71E-09 | rheumatic disease | 2.52E-28 |
| damage of bone | 2.31E-07 | arthritis | 1.34E-27 |
| Dupuytren contracture | 5.88E-07 | rheumatoid arthritis | 8.06E-25 |
| idiopathic pulmonary fibrosis | 5.00E-05 | Dupuytren contracture | 1.10E-16 |
| rheumatic disease | 1.23E-04 | osteoarthritis | 4.18E-15 |
| arthritis | 2.53E-04 | systemic lupus erythematosus | 1.03E-09 |
| ankylosing spondylitis | 7.92E-04 | ankylosing spondylitis | 1.65E-07 |
| rheumatoid arthritis | 1.85E-03 | juvenile rheumatoid arthritis | 2.11E-07 |
| systemic juvenile idiopathic arthritis | 1.93E-03 | Ehlers-Danlos syndrome, type I | 1.60E-06 |
|  |  | polyarticular juvenile rheumatoid arthritis | 3.72E-06 |
|  |  | Ehlers-Danlos syndrome | 4.94E-06 |
