# Supplementary material for: Pathway Analysis of Genetic Factors Associated with Spontaneous Preterm Birth and Pre-Labor Preterm Rupture of Membranes
Source: PLoS One. 2014 Sep 29;9(9):e108578. doi: 10.1371/journal.pone.0108578 (PMC4181300; doi:10.1371/journal.pone.0108578)
Supplement: Table S5 — Inflammatory response in PPROM and sPTB. Diverse lists of responses are shown for two phenotypes. (DOC) [file pone.0108578.s005.doc]

**Supplementary Table 5. Inflammatory response in PPROM and sPTB**

Diverse lists of responses are shown for two phenotypes.

| **PPROM** |  | **sPTB** |  |
| --- | --- | --- | --- |
| **Functions Annotation** | **p-Value** | **Functions Annotation** | **p-Value** |
| inflammation of organ | 2.05E-14 | rheumatic disease | 2.52E-28 |
| nephritis | 5.79E-09 | arthritis | 1.34E-27 |
| aggregation of blood platelets | 8.04E-08 | rheumatoid arthritis | 8.06E-25 |
| glomerulonephritis | 1.02E-07 | acne | 1.83E-19 |
| infiltration by macrophages | 4.04E-07 | hepatitis | 3.36E-16 |
| cell movement of phagocytes | 5.01E-07 | osteoarthritis | 4.18E-15 |
| focal glomerulonephritis | 2.52E-06 | viral hepatitis | 1.75E-14 |
| migration of phagocytes | 3.94E-06 | inflammatory bowel disease | 2.13E-13 |
| thin basement membrane disease | 5.30E-06 | acute respiratory distress syndrome | 6.23E-13 |
| inflammation of lung | 1.01E-05 | chronic hepatitis | 7.00E-13 |
| proliferation of phagocytes | 1.29E-05 | Crohn's disease | 2.90E-12 |
| quantity of phagocytes | 1.50E-05 | dermatitis | 5.73E-12 |
| invasion of blood-derived mast cells | 3.17E-05 | esophagitis | 1.01E-11 |
| activation of phagocytes | 3.97E-05 | asthma | 1.04E-11 |
| idiopathic pulmonary fibrosis | 5.00E-05 | hepatitis C | 1.16E-10 |
| activation of dendritic cells | 6.62E-05 | primary biliary cirrhosis | 2.79E-10 |
| cell movement of neutrophils | 7.62E-05 | Kawasaki's disease | 4.32E-10 |
| Alport's syndrome | 7.90E-05 | chronic hepatitis C | 4.55E-10 |
| fever | 7.90E-05 | systemic lupus erythematosus | 1.03E-09 |
