# Supplementary material for: Pathway Analysis of Genetic Factors Associated with Spontaneous Preterm Birth and Pre-Labor Preterm Rupture of Membranes
Source: PLoS One. 2014 Sep 29;9(9):e108578. doi: 10.1371/journal.pone.0108578 (PMC4181300; doi:10.1371/journal.pone.0108578)
Supplement: Table S6 — Comparison of molecules/genes in networks. All molecules (focus genes (in bold) and new molecules identified by IPA) in each of the significant networks. Molecules common to the PPROM and sPTB networks (B and C) and those that are unique to PPROM (in neither of the two sPTB networks) are listed. (DOC) [file pone.0108578.s006.doc]

**Supplementary Table 6. Comparison of molecules/genes in networks**

All molecules (focus genes (in bold) and new molecules identified by IPA) in each of the significant networks. Molecules common to the PPROM and sPTB networks (B and C) and those that are unique to PPROM (in neither of the two sPTB networks) are listed.

| **Network A : PPROM all molecules** | **ANG,AGT, CD14, COL1A1, COL4A3, CSF1, CSPG2 (VCAN), F3, FGF4, IFNGR2, IGF1, IGF1R, IL1A, IL6R, LIPC, LPL, MMP1, MMP10, NOS2A, NOS3, PLAT, PTGER1, REN, TBXAS1, TIMP2, TNFA, TNFRSF6, TNFR2, VWF.** ABCC1, ADIPOR1, AKT, ALDH1A3, Ap1, ARHGEF4, B4GALT5, BDKRB1, BMX, BTC, BTLA, CASP8AP2, CCBP2, CCL21, CCL27, CD300LF, CD44, CDH13, CEBP, CLEC11A, CNKSR1, CXCL16, ECE1, EGLN2, ERK, ERK1/2, ESTROGEN RECEPTOR, ETS1, FGA, FXN, G0S2, Gm-csf, GNB4, Hcg, HDL, Hsp27, HSPE1, HTR4, IFN gamma, IKK (complex), IL6, IL8, IL25, IL1RL1, IL36A, IL36G, IRAK2, ITGAV, JNK, KCNJ4, KSR1, KSR2, LDL, LILRA5, LITAF,LPAR3, lymphotoxin-alpha1-beta2, MAP4, MAP3K10, MAP4K4, MAPK, MED28, mir-26, mir-192, miR-219-5p (miRNAs w/seed GAUUGUC), MKK3/6, MMP14, NEU1,NEU3, NFkB (complex), NKRF, NLRP2, NOD1, P2RY6, P38 MAPK, Pdgf (complex), PI3K (complex), PKN3, PPARG, PP2R5E, PPT1, PRODH, PTPN12, RAP1A, RFTN1, RGS20, RNF34, S100A12, S100B, S1PR2, SAA, SCAVENGER RECEPTOR CLASS A, SEMA3C, SGK3, SLC20A1, SMG1, SNCG, SP7, TBC1D4, TFAM, TFF2, TM4SF1, TMEM9B, TNIP3, TP53, TP53RK, USP43, Vegf, VRK2, ZC3H18, ZNF24. |
| --- | --- |
| **Network B: sPTB All molecules** | **ADRB2, AGT, CCL2, CCL8, COL1A2, COL5A1, COL5A2, CTLA4, CYP19A1, DHFR, F3, FGF1, F5, F7, HSPA1B, HSPG2, IFNG, IGF1, IL10, IL10RA, IL10RB, IL12B, IL15, IL18, IL1A, IL1B, IL1R1, IL1R2, IL1RAP, IL1RN, IL2RA, IL2RB, IL4, IL4R, IL5, IL6, IL6R, IL8, KL, LTF, MMP1, MMP10, MMP2, MMP3, MMP8, MMP9, MTHFD1, NFKB1, NFKBIB, NOD2, NOS3, NR3C1, PGRMC1, PLA2G4A, PLAT, PON1, PTGS1, SCNN1A, SELE, SERPINE1, THBS4, THPO, TIMP2, TIMP3, TLR2, TLR7, TNFR1, TNFR2, TSHR, VEGFA, VEGFC.** Akt, AMPK, Ap1, BCR (complex), c1Q, CD3, Cdk, Cebp, Cplan2, Creb, E2f,Eotaxin, ERK, ERK1/2, estrogen receptor, Fcer1, Fcgr3, Fibrinogen, Focal adhesion kinase, FSH, Gm-csf, Gsk3, Hcg, Hdac, haemoglobin, Histone h3, Histone h4, HLA-DQ, HLA-DR, Hsp27, Hsp70, Hsp90, Ige, IgG1, IgG, Ikb, IL1, IL23, IL-1R, IL12 (complex), IL12 (family), Immunoglobulin, Interferon alpha, Jnk, LDL, Lh, limphotoxin-alpha1-beta2, MAP2K1/2, Mapk, MHC Class II (complex), N-cor, NFkB (complex), p38 MAPK, p85 (pik3r) ,Pdgf (complex), PDGF BB, PI3K (complex), PI3K (family), Pkc(s), Ras, Ras homolog, Rb, RNA polymerase II, SAA, STAT5a/b, TCR, TGF beta, TLR, VEGF. |
| **Network C: sPTB All molecules** | **AGTR1, CBY1, COL1A2, COL3A1, COL4A3, COL5A1, CRHR2, EPHX1, HSPA4, HSPA6, HSPA1L, OXTR, POMC, PTGER3, REN, TBXAS1,UGT1A1.** ADA, ADD2, AEN, AKAP12, ANPEP, ARHGEF12, AXIN1, BAX, BCLAF1, BDKRB2, BHLHE40, BIK, BLZF1, BTG1, BUB1, CCNL2, CDKN3, CEBPA, CENPF, CITED2, CKS2, COL7A1, CRIP2, CSF3R, CTNNA2, CTNNB1, CUL5, DKK1, ENPP1, EPHB2, ERCC1, FBN1, FGF9, FUBP1, GABPA, GBP1, GLIPR1, glutathione peroxidise, GPX2, H1FX, HAS3, Hat, HINT1, Histone H1, Histone h3, HOXA11, IFI16, IFI27, IGFBP5, IGFBP6, IGFBP7, ITGAV, KDM5A, KIF23, LBR, LEP, MAPK1, MAPK13, mir-24, mir-34, mir-224, miR-224-5p, miR-24-3p, MT1L, MYBL1, NAA10, NAP1L1, NCAPD2, NCAPG, NEK2, PARK7, PFKFB3, PKM2, POLD2, POLE2, POSTN, PPARG, PPM1A, PPP1R13L, PPP3CA, PRIM1, PRKAA1, PRODH, PTPN12, PTPRZ1, RAB31, RAD23A, ROR2, RPL11, SECTM1, SEMA3C, SERPINB5, SERPINF1, SF3B3, SFN, SFRP1, SGPL1, SIAH1, SLC5A8, Smad, SMC2, SOX4, SPOCK1, TBL1X, TGFB1, TIP60, TMSB15A, TP63, TP53, TP53AIP1, TPT1, TRD@, TRIB2, TRIM22, TUBA1A, UIMC1,USP11, VCAN, VRK1, WDHD1, YWHAE, ZBTB5, ZYX. |
| **Molecules common to PPROM & sPTB network B** | **33 AGT, F3, IGF1, IL1A, IL6R, MMP1, MMP10, NOS3, PLAT, TIMP2, TNFR2.** Akt, Ap1, Cebp, ERK, ERK1/2, estrogen receptor, Gm-csf, Hcg, Hsp27, Jnk, LDL, Lh, limphotoxin-alpha1-beta2, MAPK, NFkB (complex), p38 MAPK, Pdgf (complex), PI3K (complex), SAA, VEGF. IL6, IL8, |
| **Molecules common to PPROM & sPTB network C** | **10 COL4A3, REN, TBXAS1,**ITGAV, PPARG, PRODH, PTPN12, SEMA3C, TP53 VCAN |
| **Molecules unique to PPROM (not in network B or C)** | **101 ANG, CD14, COL1A1, CSF1, FGF4, IFNGR2, IGF1R, LIPC, LPL, NOS2A, PTGER1, TBXAS1, TNFA, TNFRSF6, VWF.**ABCC1, ADIPOR1, ALDH1A3, ARHGEF4, B4GALT5, BDKRB1, BMX, BTC, BTLA, CASP8AP2, CCBP2, CCL21, CCL27, CD300LF, CD44, CDH13, CLEC11A, CNKSR1, CXCL16, ECE1, EGLN2, ETS1, FGA, FXN, G0S2, GNB4, HDL, Hsp27, HSPE1, HTR4, Ifn gamma, IKK (complex), IL25, IL1RL1, IL36A, IL36G, IRAK2, KCNJ4, KSR1, KSR2, LILRA5, LITAF,LPAR3, MAP4, MAP3K10, MAP4K4, MED28, mir-26, mir-192, miR-219-5p (miRNAs w/seed GAUUGUC), MKK3/6, MMP14, NEU1,NEU3, NKRF, NLRP2, NOD1, P2RY6, P38, PKN3, PP2R5E, PPT1, RAP1A, RFTN1, RGS20, RNF34, S100A12, S100B, S1PR2, SCAVENGER RECEPTOR CLASS A, SGK3, SLC20A1, SMG1, SNCG, SP7, TBC1D4, TFAM, TFF2, TM4SF1, TMEM9B, TNIP3, TP53RK, USP43, VRK2, ZC3H18, ZNF24. |
| **Molecules unique to SPTB Networks B and C (not in Network A)** | **236 ADRB2, CCL2, CCL8, COL1A2, COL5A1, COL5A2, CTLA4, CYP19A1, DHFR, FGF1, F5, F7, HSPA1B, HSPG2, IFNG, IL10, IL10RA, IL10RB, IL12B, IL15, IL18, IL1B, IL1R1, IL1R2, IL1RAP, IL1RN, IL2RA, IL2RB, IL4, IL4R, IL5, KL, LTF, MMP2, MMP3, MMP8, MMP9, MTHFD1, NFKB1, NFKBIB, NOD2, NR3C1, PGRMC1, PLA2G4A, PON1, PTGS1, SCNN1A, SELE, SERPINE1, THBS4, THPO, TIMP3, TLR2, TLR7, TNFR1, TSHR, VEGFA, VEGFC. AGTR1, CBY1, COL3A1, CRHR2, EPHX1, HSPA4, HSPA6, HSPA1L, OXTR, POMC, PTGER3, UGT1A1.** AMPK, BCR (complex), c1Q, CD3, Cdk, Cplan2, Creb, E2f,Eotaxin, Fcer1, Fcgr3, Fibrinogen, Focal adhesion kinase, FSH, Gsk3, Hdac, haemoglobin, Histone h3, Histone h4, HLA-DQ, HLA-DR, Hsp70, Hsp90, Ige, IgG1, IgG, Ikb, IL1, IL23, IL-1R, IL12 (complex), IL12 (family), Immunoglobulin, Interferon alpha, Lh, MAP2K1/2, MHC Class II (complex), N-cor, , p85 (pik3r) ,Pdgf (complex), PI3K (family), Pkc(s), Ras, Ras homolog, Rb, RNA polymerase II, STAT5a/b, TCR, TGF beta, TLR. ADA, ADD2, AEN, AKAP12, ANPEP, ARHGEF12, AXIN1, BAX, BCLAF1, BDKRB2, BHLHE40, BIK, BLZF1, BTG1, BUB1, CCNL2, CDKN3, CEBPA, CENPF, CITED2, CKS2, COL7A1, CRIP2, CSF3R, CTNNA2, CTNNB1, CUL5, DKK1, ENPP1, EPHB2, ERCC1, FBN1, FGF9, FUBP1, GABPA, GBP1, GLIPR1, glutathione peroxidise, GPX2, H1FX, HAS3, Hat, HINT1, Histone H1, HOXA11, IFI16, IFI27, IGFBP5, IGFBP6, IGFBP7, KDM5A, KIF23, LBR, LEP, MAPK1, MAPK13, mir-24, mir-34, mir-224, miR-224-5p, miR-24-3p, MT1L, MYBL1, NAA10, NAP1L1, NCAPD2, NCAPG, NEK2, PARK7, PFKFB3, PKM2, POLD2, POLE2, POSTN, PPM1A, PPP1R13L, PPP3CA, PRIM1, PRKAA1, PTPRZ1, RAB31, RAD23A, ROR2, RPL11, SECTM1, SERPINB5, SERPINF1, SF3B3, SFN, SFRP1, SGPL1, SIAH1, SLC5A8, Smad, SMC2, SOX4, SPOCK1, TBL1X, TGFB1, TIP60, TMSB15A, TP63, TP53AIP1, TPT1, TRD@, TRIB2, TRIM22, TUBA1A, UIMC1,USP11, VRK1, WDHD1, YWHAE, ZBTB5, ZYX. |
